# Perceived COVID-19 threat, perceived healthcare system inequities, personal experiences of healthcare discrimination and their associations with COVID-19 preventive behavioral intentions among college students in the U.S.

**DOI:** 10.1186/s12889-022-14438-5

**Published:** 2022-12-31

**Authors:** Juliana S. Sherchan, Jessica R. Fernandez, Shan Qiao, Arie W. Kruglanski, Allana T. Forde

**Affiliations:** 1 Division of Intramural Research, National Institute on Minority Health and Health Disparities, National Institutes of Health, Bethesda, MD USA; 2 Department of Psychology, University of Maryland, College Park, MD USA; 3 Health Promotion, Education, and Behavior, Arnold School of Public Health, University of South Carolina, Columbia, SC USA

**Keywords:** College/university students, COVID-19 prevention, COVID-19 threat, Healthcare discrimination, Latent class, Perceived U.S. healthcare system inequities, Race/ethnicity

## Abstract

College students are often reluctant to follow U.S. preventive guidelines to lower their risk of COVID-19 infection, despite an increased risk of transmission in college settings. Prior research suggested that college students who perceived greater COVID-19 severity and susceptibility (i.e., COVID-19 threat) were more likely to engage in COVID-19 preventive behaviors, yet there is limited research examining whether perceived COVID-19 threat, perceived U.S. healthcare system inequities, and personal experiences of healthcare discrimination collectively influence college students’ COVID-19 preventive behaviors. This study identified latent classes of perceived COVID-19 threat, perceived U.S. healthcare system inequities, and personal experiences of healthcare discrimination, examined whether latent classes were associated with COVID-19 preventive behavioral intentions, and assessed whether latent class membership varied across racial/ethnic groups.

Students from the University of Maryland, College Park (*N* = 432) completed the *Weighing Factors in COVID-19 Health Decisions* survey (December 2020-December 2021). Latent class analysis identified latent classes based on perceived COVID-19 threat, perceived U.S. healthcare system inequities, and personal experiences of healthcare discrimination. Regression analyses examined associations between the latent classes and COVID-19 preventive behavioral intentions (i.e., social distancing, mask-wearing, COVID-19 vaccination) and whether latent class membership varied across racial/ethnic groups.

Students in Latent Class 1 (27.3% of the sample) had high perceived COVID-19 threat and U.S. healthcare system inequities and medium probability of experiencing personal healthcare discrimination. Students in Latent Class 1 had higher social distancing, mask-wearing, and vaccination intentions compared to other latent classes. Compared to Latent Class 4 (reference group), students in Latent Class 1 had higher odds of identifying as Hispanic or Latino, Non-Hispanic Asian, Non-Hispanic Black or African American, and Non-Hispanic Multiracial versus Non-Hispanic White.

Latent classes of higher perceived COVID-19 threat, perceived U.S. healthcare system inequities, and personal experiences of healthcare discrimination were associated with higher COVID-19 preventive behavioral intentions and latent class membership varied across racial/ethnic groups. Interventions should emphasize the importance of COVID-19 preventive behaviors among students who perceive lower COVID-19 threat.

**Supplementary Information:**

The online version contains supplementary material available at 10.1186/s12889-022-14438-5.

In the early months of 2020, U.S. public health officials began recommending the use of face masks and social distancing to provide protection against the highly transmissible COVID-19 virus followed by an unprecedented production of multiple vaccines [1]. Despite these efforts, public reluctance to follow guidelines on social distancing, mask-wearing, and COVID-19 vaccination slowed the containment of the COVID-19 virus [2, 3] and the COVID-19 pandemic resulted in over 900,000 deaths in the U.S. between March 2020 and March 2022 [4]. A simulation study of 10.5 million adults in North Carolina found that lack of adherence to COVID-19 preventive behaviors resulted in 8,000 preventable deaths, [5] which suggests that hundreds of thousands of U.S. deaths may have been prevented with greater adherence to public health guidelines.

The reluctance to adhere to COVID-19 countermeasures was particularly evident in younger populations [6], including college students who were often reluctant to follow guidelines on mask-wearing and social distancing [7]. Moreover, widespread transmission of the COVID-19 virus among large clusters of people, such as those on college campuses, posed health concerns throughout the U.S. [8]. A simulation model of a U.S. college campus conducted prior to the fall 2020 semester revealed that a lack of recommended mask adoption and social distancing on campus resulted in considerable spread of COVID-19 infection and increased risk of death from COVID-19 among college students [9]. These simulated risks were consistent with observed COVID-19 case rates and COVID-19-related deaths among college students during the 2020–2021 academic year (i.e., COVID-19 case rates among college students in the U.S. rose to over 700,000 by the end of the 2020–2021 academic year and over 100 COVID-19-related deaths occurred on campuses throughout the country [10]). In addition, COVID-19-related deaths doubled in counties with large college populations [2]. Together, these findings suggest the importance of college students following COVID-19 preventive guidelines to lower case rates and the risk of death on college campuses and surrounding communities. Consequently, cases of low prevalence of preventive behaviors among college students and the potential for college campuses to become hot spots for rapid viral transmission highlight the urgent need to better understand the factors influencing COVID-19 preventive behaviors among populations in college settings [11].

Various underlying health-related perceptions that are broadly characterized as drivers and barriers have been posited to influence preventive behaviors [12]. Specifically, previous studies found that perceptions of higher disease severity and susceptibility to infection can serve as drivers of preventive health behaviors [13], whereas perceptions of healthcare inequities (e.g., perceptions of fairness in the healthcare system) and experiences of healthcare discrimination [14] can serve as barriers to preventive health behaviors. Recent studies found that perceived COVID-19 severity and susceptibility [15], perceived U.S. healthcare system inequities [16], and personal experiences of healthcare discrimination [17] varied in the general population.

There is also emerging evidence that these health-related perceptions vary among college students, with a growing number of studies focused on perceived COVID-19 severity and susceptibility [18, 19]. However, while there is some research on college students’ perceptions of the U.S. healthcare system [20, 21] and personal experiences of healthcare discrimination [22], the studies on these topics are limited. Specifically, studies have yet to examine additional factors related to the healthcare system, including college students’ perceptions of treatment, access, and/or distribution of COVID-19 health services and racial/ethnic differences in these perceptions.

Recent studies revealed associations between college students’ perceptions of COVID-19 severity and susceptibility and certain COVID-19 preventive health behaviors such as mask-wearing, social distancing, and/or COVID-19 vaccination, which were policies adopted by most U.S. colleges at the time of each study [7, 18]. Despite findings showing associations between perceptions of the healthcare system and personal experiences of healthcare discrimination with COVID-19 preventive behaviors in the general population [23, 24], there is a lack of research examining these associations among college students. Furthermore, preventive behaviors such as mask-wearing, social distancing, and COVID-19 vaccination may vary in their protection against the COVID-19 virus [25], perceived behavioral effort [26], and the extent to which the preventive behaviors involve the healthcare system. Therefore, it is possible that health perceptions at the individual level (i.e., perceived COVID-19 severity and susceptibility) and system level (i.e., perceived U.S. healthcare system inequities and personal experiences of healthcare discrimination) may have differential impacts on these behaviors.

To date, no studies have examined whether individual risk perceptions, perceptions of the healthcare system, and personal experiences of healthcare discrimination (“complex health-related perceptions”) collectively influence various COVID-19 behaviors. One approach to studying the collective influence of these perceptions includes latent class analyses, a modeling technique used to identify patterns of responses to a set of observed measures [27]. In the context of complex health-related perceptions, these patterns could represent subgroups or “latent classes” of individuals who respond similarly to each of the health-related perceptions and certain patterns of responses might influence COVID-19 preventive behavioral intentions. One class, for example, could include high perceived COVID-19 threat, low perceived healthcare system inequity, and low personal experiences of healthcare discrimination, which might result in high preventive health behavioral intentions due to lower perceived barriers to healthcare (e.g., lower anticipated discrimination) and greater trust in the healthcare system.

Moreover, there is limited research examining racial/ethnic differences in college students’ health-related perceptions, even though individuals from minoritized racial/ethnic groups have been most affected by the COVID-19 pandemic (e.g., higher rates of death among African American, Native American, and Latinx American individuals compared to Non-Hispanic White American individuals [28]). In addition, given that students from minoritized racial/ethnic groups are more likely to have prior experiences of healthcare discrimination and could be more attuned to racial/ethnic disparities in the U.S. healthcare system, it is possible that these patterns of complex health-related perceptions could partially explain disparities in COVID-19 outcomes among college students.

The present study sought to address these gaps in the literature by conducting a latent class analysis among a sample of college students in the U.S. to identify latent classes using indicators of perceived COVID-19 threat, perceived U.S. healthcare system inequities, and personal experiences of healthcare discrimination, examine whether these latent classes were associated with COVID-19 preventive behavioral intentions, and assess racial/ethnic differences in latent class membership.

## Methods

### Data source

Data for this study was collected from the *Weighing Factors in COVID-19 Health Decisions* survey conducted from December 2020 to December 2021 at the University of Maryland, College Park (UMD). The survey period was conducted over three semesters to meet a target sample size of 400 participants. Themes within the survey included COVID-19 preventive behavioral intentions (25 items), perceptions of COVID-19 (10 items), attitudes about antibiotics, vaccines, and general trust in other people and the government (8 items), personal experiences with racism and discrimination (42 items), use of health care (18 items), health history (3 items), and demographic information (18 items). The survey was self-administered using Qualtrics software and participants were eligible if they were UMD college students and 18 years of age and older. Informed consent was obtained from all participants prior to participation using an electronic consent form, and study procedures were approved by the UMD Institutional Review Board. Four hundred and ninety-one participants consented, completed at least a portion of the survey, and received university credit for their anonymous participation. Survey responses were excluded from the analysis if they were incomplete (*n* = 38) or included small samples for specific sociodemographic categories (*n* = 21). The final sample included 432 participants.

### Measures

#### Latent class indicators

Six items were used as indicators to generate the latent classes (i.e., referred to as latent class health-related perceptions in the present study) (Table S1). The indicators captured perceived COVID-19 severity and susceptibility (i.e., collectively labeled “perceived COVID-19 threat”), perceived U.S. healthcare system inequities, and personal experiences of healthcare discrimination. Consistent with terms from prior Health Belief Model studies [29, 30], perceived severity of COVID-19 was measured using the item “You believe COVID-19 is serious and life threatening” and perceived susceptibility to COVID-19 was measured with the item “You are concerned about contracting COVID-19”. Response options for both items included “Very true,” “Somewhat true,” and “Not true.” Responses were dichotomized with “Somewhat true” and “Not true” coded as 0 and “Very true” coded as 1.

Perceived U.S. healthcare system inequities included three items on the perceived treatment of COVID-19 patients from minoritized racial/ethnic groups, access to COVID-19 testing for minoritized racial/ethnic groups compared to White individuals, and distribution of the COVID-19 vaccine across racial/ethnic groups [31]. Perceived treatment of COVID-19 patients from minoritized racial/ethnic groups was measured using the item “How often have racial and ethnic minority patients with COVID-19 been treated unfairly by the U.S. healthcare system because of their race or ethnicity?”. Response options included “Very often,” “Somewhat often”, and “Never”, and were dichotomized for the analysis with “Never” coded as 0 and “Somewhat often” and “Very often” coded as 1. Perceived access to COVID-19 testing for minoritized racial/ethnic groups was measured using the item “How true is it that racial and ethnic minority groups have less access to COVID-19 testing compared to Whites?”. Response options included “Very true,” “Somewhat true,” and “Not true” and were dichotomized for the analysis with “Not true” coded as 0 and “Somewhat true” and “Very true” coded as 1. Perceived distribution of the COVID-19 vaccine across racial/ethnic groups was measured using the item “How confident are you that the COVID-19 vaccine will be distributed fairly across racial and ethnic groups?”. Response options included “Very confident”, “Somewhat confident”, and “Not confident” and were dichotomized for the analysis with “Very confident” coded as 0 and “Somewhat confident” and “Not confident” coded as 1.

Personal experiences of healthcare discrimination were measured using a 7-item modified version of the Everyday Discrimination Scale for healthcare settings (i.e., how often participants encountered various situations when receiving health care such as “Treated with less respect than other people” or “Felt like a doctor or nurse was not listening to what you were saying”) [32, 33]. The response options were averaged (i.e., “Never” = 0, “Once” = 1, “2–3 times” = 2, “4 times or more” = 3) and used to dichotomize the personal experiences of healthcare discrimination item, with mean scores of 0 representing no experiences of healthcare discrimination and mean scores greater than 0 representing experiences of healthcare discrimination.

#### COVID-19 preventive behavioral intentions

Three items were used to capture COVID-19 preventive behavioral intentions. Participants were asked to review three separate scenarios (presented in random order) assessing how likely they would be to social distance, wear a mask in public, and receive the COVID-19 vaccine (Table S2). Response options for each behavior were measured on a 6-point scale from “Extremely unlikely,” to “Extremely likely.” All three items were treated as separate continuous outcomes in the analysis.

#### Race/ethnicity and sociodemographic covariates

Participants self-identified their race/ethnicity in two separate items. Participants were asked to self-identify their race by selecting all that applied from the U.S. Census categories of “American Indian or Alaska Native”, “Asian”, “Native Hawaiian or Other Pacific Islander”, “Black or African American”, “White”, and “Other” (with the option to specify another race). Participants were asked to self-identify their Hispanic ethnicity by selecting “Hispanic or Latino” or “Not Hispanic or Latino.” The race/ethnicity items were combined into the following categories for use in the analysis: “Hispanic or Latino”, “Non-Hispanic Asian”, “Non-Hispanic Black or African American”, “Non-Hispanic Multiracial” and “Non-Hispanic White”. Participants who self-identified as Non-Hispanic Other Race (*n* = 12) were not included in the analysis due to the small sample size. No participants identified as Non-Hispanic Native Hawaiian or Other Pacific Islander or Non-Hispanic American Indian or Alaska Native. Participants were asked to self-identify their gender as male, female, transgender male, transgender female, gender non-conforming, or gender “not listed.” Participants’ gender identity of male versus female was included as a categorical covariate (participants who identified as transgender (*n* = 1), gender non-conforming (*n* = 6), or indicated their gender was “not listed” (*n* = 3) were not included in the analysis due to small sample sizes). Participants’ household income (i.e., $0–49,999, $50,000–99,999, ≥ $100,000) and age in years were included as categorical and continuous covariates, respectively.

### Analyses

Descriptive statistics were calculated to assess the sociodemographic characteristics of the study population. Chi-square and Analysis of Variance tests were used to examine group differences in the sociodemographic characteristics of the study population across race/ethnicity. These analyses were conducted using R version 4.1.2. A latent class analysis was conducted in M*plus* Version 8.6 [34] to identify students’ latent classes from perceived COVID-19 threat, perceived U.S. healthcare system inequities, and personal experiences of healthcare discrimination, examine whether latent classes were associated with COVID-19 preventive behavioral intentions, and assess racial/ethnic differences in latent class membership (adjusting for age, gender, and household income).

#### Identification of latent class health-related perceptions

Latent class models were estimated using the six health perception indicators and robust maximum likelihood (MLR) estimation. A stepwise model comparison approach (1-class, 2-class, 3-class) was applied to select the best fitting solution based on the sample-size adjusted Bayesian Information Criterion (SA-BIC), entropy, and likelihood ratio tests. In the final step of model selection, direct effects of the sociodemographic covariates on the latent class indicators were assessed using model comparisons to test for any potential sociodemographic differences in interpreting the latent class indicators (i.e., measurement invariance) [35].

#### Associations between latent class health-related perceptions and COVID-19 preventive behavioral intentions

Linear regression was used to examine the association between the latent class health-related perceptions and intention to carry out each of the three COVID-19 preventive behaviors (i.e., social distancing, mask-wearing, COVID-19 vaccination). Each of the COVID-19 preventive behavioral intentions was regressed on latent class membership using the Bolck, Croons, & Hagenaars (BCH) method to assign latent class membership while accounting for classification uncertainty (i.e., individuals’ fractional probabilities of membership in more than one class) [36–38]. Each regression model was also adjusted for sociodemographic covariates on the COVID-19 preventive behavioral intentions as well as the survey completion date (i.e., the month in which participants completed the study), which was included as a continuous variable. Pairwise comparisons using Wald tests were used to assess differences in the predicted intercept value of each of the COVID-19 preventive behavioral intentions across the latent classes.

#### Racial/ethnic differences in latent class membership

Multinomial logistic regression was used to examine racial/ethnic differences in latent class membership. Latent class membership was regressed on race/ethnicity, adjusting for age, gender, household income and survey completion date. Odds ratios (OR) with 95% confidence intervals (CI) were used to assess the conditional probabilities of racial/ethnic group membership within each latent class (compared to the reference group of Non-Hispanic White) with all other covariates present.

## Results

Most students self-identified as Non-Hispanic White (49.5%), were on average 19.3 years of age, were female (75.0%), and had more than $100,000 (53.9%) in household income (Table 1). Significant differences in gender (*p* < 0.01) and household income (*p* < 0.01) were observed across race/ethnicity. Students who identified as Non-Hispanic White and Hispanic or Latino had the highest proportions of females (81.8% and 78.0%, respectively) and students who identified as Non-Hispanic Multiracial had the lowest proportion of females (46.7%). Students who identified as Non-Hispanic Multiracial and Non-Hispanic White had the highest proportion of students with a household income of ≥ $100,000 (70.0% and 64.0%, respectively), whereas students who identified as Non-Hispanic Black or African American and Hispanic or Latino had the lowest proportion of students with a household income of ≥ $100,000 (26.5% and 38.0%, respectively).

**Table 1:** Sociodemographic characteristics of the study population (*N* = 432)

	**Total Population** (*N*=432)	**Hispanic or Latino** **(*n* = 50)**	**Non-Hispanic Asian** **(*n* = 89)**	**Non-Hispanic Black or African American** **(*n* = 49)**	**Non-Hispanic Multiracial** **(*n* = 30)**	**Non-Hispanic White** **(*n* = 214)**	***p*-value**
	n (%) or Mean (± SD)	n (%) or Mean (± SD)	n (%) or Mean (± SD)	n (%) or Mean (± SD)	n (%) or Mean (± SD)	n (%) or Mean (± SD)	
**Age, y**	19.3 (± 1.3)	19.0 (± 1.2)	19.4 (± 1.4)	19.4 (± 1.4)	19.2 (± 0.9)	19.4 (± 1.4)	0.50
Min–Max	18–27	18–23	18–24	18–23	18–21	18–27	
**Gender**							< 0.01
Male	108 (25.0)	11 (22.0)	35 (39.3)	16 (32.7)	7 (23.3)	39 (18.2)	
Female	324 (75.0)	39 (78.0)	54 (60.7)	33 (67.3)	23 (46.7)	175 (81.8)	
**Household Income**							< 0.01
$ < 50,000	68 (15.8)	14 (28.0)	21 (23.6)	14 (28.6)	2 (6.7)	17 (8.0)	
$50,000–99,999	131 (30.3)	17 (34.0)	25 (28.1)	22 (44.9)	7 (23.3)	60 (28.0)	
> $100,000	233 (53.9)	19 (38.0)	43 (48.3)	13 (26.5)	21 (70.0)	137 (64.0)	
**COVID-19 Severity**							0.31
Not severe to somewhat severe	91 (21.4)	6 (12.0)	19 (21.6)	9 (18.4)	5 (16.7)		
Very severe	335 (78.6)	44 (88.0)	69 (78.4)	40 (81.6)	25 (83.3)		
Missing*	6	0	1	0	0	5	
**COVID-19 Susceptibility**							<0.01
Not susceptible to somewhat susceptible	259 (60.1)	22 (44.0)	45 (50.6)	25 (51.0)	14 (46.7)	153 (71.8)	
Very susceptible	172 (39.9)	28 (56.0)	44 (49.4)	24 (49.0)	16 (53.3)	60 (28.2)	
Missing	1	0	0	0	0	1	
**Healthcare Discrimination**							<0.01
No discrimination	311 (72.3)	30 (60.0)	56 (63.6)	17 (34.7)	23 (76.7)	185 (86.9)	
Discrimination	119 (27.7)	20 (40.0)	32 (36.4)	32 (65.3)	7 (23.3)	28 (13.1)	
Missing	2	0	1	0	0	1	
**Unfair Treatment**							0.57
Fair	48 (11.2)	5 (10.0)	13 (14.8)	4 (8.2)	5 (16.7)	21 (9.9)	
Somewhat fair to unfair	381 (88.8)	45 (90.0)	75 (85.2)	45 (91.8)	25 (83.3)	191 (90.1)	
Missing	3	0	1	0	0	2	
**Less Access**							0.21
Not true (no difference in access)	77 (17.9)	6 (12.0)	22 (25.0)	11 (22.4)	5 (16.7)	33 (15.4)	
Somewhat to very true (less access)	354 (82.1)	44 (88.0)	66 (75.0)	38 (77.6)	25 (83.3)	181 (84.6)	
Missing	1	0	1	0	0	0	
**Unfair Distribution**							0.37
Very confident	58 (13.4)	6 (12.0)	10 (11.2)	3 (6.1)	4 (13.3)	35 (16.4)	
Somewhat to not confident	374 (86.6)	44 (88.0)	79 (88.8)	46 (93.9)	26 (86.7)	179 (83.6)	
**Social Distancing**	4.7 (± 1.4)	5.0 (± 1.2)	5.0 (± 1.1)	5.1 (± 1.1)	4.8 (± 1.3)	4.4 (± 1.5)	<0.01
Min–Max	1–6	1–6	1–6	2–6	1–6	1–6	
**Mask-Wearing**	5.4 (± 1.1)	5.6 (± 0.9)	5.6 (± 1.0)	5.6 (± 0.9)	5.8 (± 0.6)	5.2 (± 1.2)	<0.01
Min–Max	1–6	1–6	1–6	1–6	4–6	1–6	
**COVID-19 Vaccination**	5.6 (± 1.1)	5.6 (± 1.0)	5.6 (± 1.1)	5.4 (± 1.3)	5.8 (± 0.6)	5.6 (± 1.1)	0.58
Min–Max	1–6	1–6	1–6	2–6	3–6	1–6	

SD=Standard Deviation

*P*-values based on χ2 and ANOVA tests of group differences across race/ethnicity

^*^Note: The maximum likelihood estimator used in the analysis allowed for missing data on the latent class indicators

### Identification of latent class health-related perceptions

The four-class solution was selected as the best fitting model of latent classes based on model fit indices (i.e., lower SA-BIC, higher entropy, and the likelihood ratio tests) (Table S3). Model comparisons revealed a lower SA-BIC for the model without direct effects of the sociodemographic covariates on the latent class indicators, and therefore the final model did not include these direct effects. The four latent classes were labeled according to the estimated probabilities of each of the six latent class indicators (Table 2, Figure S1).

**Table 2:** Estimated probabilities of agreement with latent class indicators

	**Class 1** **High** PCT **High** PHSI **Medium** PEHD ***n* = 118 (27.3%)**	**Class 2** **Low-Medium** PCT **Medium** PHSI **Low** PEHD ***n* = 73 (16.9%)**	**Class 3** **Low-Medium** PCT **Low–High** PHSI **High** PEHD ***n* = 22 (5.0%)**	**Class 4** **Low–High** PCT **High** PHSI **Low** PEHD ***n* = 219 (50.8%)**
**Perceived COVID-19 Threat**				
COVID-19 Severity	1.00	0.61	0.47	0.76
COVID-19 Susceptibility	1.00	0.24	0.29	0.14
**Perceived U.S. Healthcare System Inequities**				
Treatment of COVID-19 Patients	0.95	0.49	0.75	1.00
Access to COVID-19 Testing	0.97	0.37	0.00	0.98
COVID-19 Vaccine Distribution	1.00	0.44	0.73	0.95
**Personal Experiences of Healthcare Discrimination**				
Healthcare Discrimination	0.39	0.00	1.00	0.24

Estimated probabilities of selecting 1 versus 0 for each dichotomously coded latent class indicator. The classes were labeled using the estimated probabilities of perceived COVID-19 threat (PCT), perceived healthcare system inequities (PHSI), and personal experiences of healthcare discrimination (PEHD) (categorized as low: < 33%, medium: 33–66%, high: > 66%)

Students in Latent Class 1 (27.3% of the sample) had *high* probabilities of perceiving COVID-19 threat, *high* probabilities of perceiving U.S. healthcare system inequities, and a *medium* probability of experiencing personal healthcare discrimination. Students in Latent Class 2 (16.9% of the sample) had *low to medium* probabilities of perceiving COVID-19 threat, *medium* probabilities of perceiving U.S. healthcare system inequities, and a *low* probability of experiencing personal healthcare discrimination. Students in Latent Class 3 (5.0% of the sample) had *low to medium* probabilities of perceiving COVID-19 threat, *low to high* probabilities of perceiving U.S. healthcare system inequities, and a *high* probability of experiencing personal healthcare discrimination. Students in Latent Class 4 (50.8% of the sample) had *low to high* probabilities of perceiving COVID-19 threat, *high* probabilities of perceiving U.S. healthcare system inequities, and a *low* probability of experiencing personal healthcare discrimination.

### Associations between latent class health-related perceptions and COVID-19 preventive behavioral intentions

Students’ intentions to social distance in public (SD), wear a mask (MSK), and receive the COVID-19 vaccine (VAX) varied across the latent class health-related perceptions (Table 3, Figure S2A-C). Latent Class 1 included students with the highest COVID-19 preventive behavioral intentions (i.e., the predicted value of the intercept) (SD: OR 5.58 [95% CI: 4.79–6.36], MSK: OR 5.74 [95% CI: 5.39–6.09], VAX: OR 5.49 [95% CI: 4.99–5.99]), followed by Latent Class 4 (SD: OR 4.83 [95% CI: 4.42–5.24], MSK: OR 5.50 [95% CI: 5.26–5.74], VAX: OR 5.39 [95% CI: 5.04–5.74]), Latent Class 2 (SD: OR 4.49 [95% CI: 3.86–5.12], MSK: OR 4.87 [95% CI: 4.43–5.31], VAX: OR 4.91 [95% CI: 4.41–5.42]), and Latent Class 3 (SD: OR 3.92 [95% CI: 3.14–4.70], MSK: OR 4.79 [95% CI: 4.20–5.38], VAX: OR 3.74 [95% CI: 3.22–5.16]). Pairwise comparisons revealed statistically significant differences (p < 0.05) in the predicted COVID-19 preventive behavioral intentions across latent classes. Students in Latent Class 1 had significantly higher intentions to social distance in public compared to students in Latent Classes 2, 3, and 4 (Wald χ2 [df=1] = 13.98, *p* < 0.01, Wald χ2 [df=1] = 9.20, *p* < 0.01, Wald χ2 [df=1] = 6.39, *p* = 0.01, respectively). Students in Latent Class 4 had significantly higher intentions to social distance in public compared to students in Latent Class 3 (Wald χ2 [df=1]=5.20, *p*=0.02). The patterns for wearing a mask and receiving the COVID-19 vaccine were similar. Students in Latent Class 1 had significantly higher intentions to wear a mask in public and receive a COVID-19 vaccine compared to students in Latent Class 2 (Wald χ2 [df=1] = 12.16, *p* < 0.01, Wald χ2 [df=1] = 7.86, *p* < 0.01, respectively) and Latent Class 3 (Wald χ2 [df=1] = 6.83, *p* < 0.01, Wald χ2 [df=1] = 6.79, *p* < 0.01, respectively). Students in Latent Class 4 also had significantly higher intentions to wear a mask in public and receive a COVID-19 vaccine compared to students in Latent Class 2 (Wald χ2 [df=1] = 7.32, *p* < 0.01, Wald χ2 [df=1] = 5.62, *p* = 0.02, respectively) and Latent Class 3 (Wald χ2 [df=1] = 5.91, *p* = 0.02, Wald χ2 [df=1] = 6.48, *p* = 0.01, respectively).

**Table 3:** Likelihood of COVID-19 preventive behavioral intentions by latent classes

	**Class 1** **High** PCT **High** PHSI **Medium** PEHD	**Class 2** **Low-Medium** PCT **Medium** PHSI **Low** PEHD	**Class 3** **Low-Medium** PCT **Low–High** PHSI **High** PEHD	**Class 4** **Low–High** PCT **High** PHSI **Low** PEHD
**Preventive Behavioral Intentions**				
Social Distancing	5.58 [4.79–6.36]	4.49 [3.86–5.12]	3.92 [3.14–4.70]	4.83 [4.42–5.24]
Mask-Wearing	5.74 [5.39–6.09]	4.87 [4.43–5.31]	4.79 [4.20–5.38]	5.50 [5.26–5.74]
COVID-19 Vaccination	5.49 [4.99–5.99]	4.91 [4.41–5.42]	3.74 [3.22–5.16]	5.39 [5.04–5.74]

Likelihood of COVID-19 preventive behavioral intentions represents predicted value of intercept, adjusting for age, gender, household income, and survey completion date. The classes were labeled using the estimated probabilities of perceived COVID-19 threat (PCT), perceived healthcare system inequities (PHSI), and personal experiences of healthcare discrimination (PEHD) (categorized as low: < 33%, medium: 33-66%, high: > 66%). 95% Confidence Intervals presented in brackets

### Racial/ethnic differences in latent class membership

Compared to students in the Latent Class 4 reference group, students in Latent Class 1 had higher odds of self-identifying as Hispanic or Latino, Non-Hispanic Asian, Non-Hispanic Black or African American, and Non-Hispanic Multiracial compared to self-identifying as Non-Hispanic White (OR: 7.51 [95% CI: 2.83–19.90], OR: 4.29 [95% CI: 1.90–9.66], OR: 4.51 [95% CI: 1.74–11.68], OR: 3.85 [95% CI: 1.37–10.82], respectively) (Table 4). Students’ race/ethnicity did not significantly vary between Latent Classes 2 and 3 compared to the Latent Class 4 reference group.

**Table 4:** Racial/ethnic differences in latent classes

	**Class 1** **High** PCT **High** PHSI **Medium** PEHD	**Class 2** **Low-Medium** PCT **Medium** PHSI **Low** PEHD	**Class 3** **Low-Medium** PCT **Low–High** PHSI **High** PEHD
**Race/ethnicity**	**Odds Ratio [95% CI]**	**Odds Ratio [95% CI]**	**Odds Ratio [95% CI]**
Hispanic or Latino	7.51 [2.83–19.90]	1.69 [0.48–5.96]	1.18 [0.22–6.37]
Non-Hispanic Asian	4.29 [1.90–9.66]	2.05 [0.87–4.82]	0.48 [0.06–3.75]
Non-Hispanic Black	4.51 [1.74–11.68]	0.52 [0.12–2.29]	2.20 [0.44–11.14]
Non-Hispanic Multiracial	3.85 [1.37–10.82]	1.21 [0.31–4.76]	1.05 [0.08–13.96]
Non-Hispanic White (reference)	-	-	-

Multinomial logistic regression model using the four-class solution (with Class 4 as the reference class). The classes were labeled using the estimated probabilities of perceived COVID-19 threat (PCT), perceived healthcare system inequities (PHSI), and personal experiences of healthcare discrimination (PEHD) (categorized as low: < 33%, medium: 33-66%, high: > 66%). Model adjusted for age, gender, household income, and survey completion date

## Discussion

This study was conducted to better understand the COVID-19 preventive behavioral intentions of college students given the high risk of COVID-19 transmission on college campuses in the U.S. Although previous studies examined college students’ preventive behavioral intentions related to perceived COVID-19 threat, the present study expanded on these studies by identifying four distinct latent class health-related perceptions among college students related to perceived COVID-19 threat, perceived U.S. healthcare system inequities, and personal experiences of healthcare discrimination. The present study also examined the associations between these latent class health-related perceptions and COVID-19 preventive behavioral intentions and assessed racial/ethnic differences in latent class membership.

Most students reported higher perceived COVID-19 severity compared to COVID-19 susceptibility, suggesting that most students had low concerns about contracting the virus despite understanding that COVID-19 is a serious illness. These findings may reflect health perceptions that are specifically related to the age of college students, whereby adolescent and young adults may have higher perceptions of invincibility [39] and lower risk perceptions due to lower rates of COVID-19 deaths among younger age groups [5]. In addition, most students perceived racial/ethnic inequities in the U.S. healthcare system which is higher than recent nationally representative studies in the general adult population [16]. These perceptions may be partially attributed to discussions of structural racism on university and college campuses, which increased on campuses across the country in 2020 [40] and may suggest perceptions of racial/ethnic inequities in the healthcare system are higher among college students compared to the general adult population. Lastly, students’ probability of experiencing personal healthcare discrimination varied across latent classes which reflects the literature on healthcare discrimination in the general adult population [41]. Responses to the latent class indicators suggest that college students may vary in their individual risk perceptions (i.e., perceived severity and susceptibility to illness) as well as perceptions of the healthcare system. Such findings are particularly important for public health, given that health behaviors are driven by multiple levels of influence [42] and college students’ perceptions of the healthcare system are understudied in the literature [18, 22].

### Health-related perceptions across latent classes

Students in Latent Class 1 reported higher perceived COVID-19 threat (specifically, perceived COVID-19 susceptibility) compared to students in all other latent classes, possibly because they were more likely to identify as Hispanic or Latino, Non-Hispanic Asian, Non-Hispanic Black or African American, and Non-Hispanic Multiracial compared to Non-Hispanic White. Given the racial/ethnic disparities in COVID-19 infection and clinical consequences (i.e., minoritized racial/ethnic populations remain more likely to contract COVID-19 and experience severe infection [43]), perceived COVID-19 severity and susceptibility among Latent Class 1 members may reflect higher perceived COVID-19 threat because of these national-level health disparities. Higher perceived COVID-19 threat among college students of minoritized racial/ethnic backgrounds is also consistent with previous literature [44]. These findings are important to the well-being of college students, given that increased perceived COVID-19 threat could exacerbate daily stressors already experienced by college students, therefore contributing to racial/ethnic differences in COVID-19 related stress [45, 46].

### COVID-19 preventive behavioral intentions across latent classes

Students in Latent Class 1 had higher intentions to social distance compared to students in all other latent classes and higher intentions to wear a mask and receive the COVID-19 vaccine compared to students in Latent Classes 2 and 3. Given that students in Latent Class 1 were more likely to belong to one of the minoritized racial/ethnic groups compared to the Non-Hispanic White group, their intentions to engage in COVID-19 preventive behaviors are partially reflective of recent national surveys of U.S. adults (i.e., Black and Latinx adults were more likely to social distance [41] and Black and Latinx males and females and Asian males were more likely to wear masks than White respondents [47]).

Consistent with prior explanations from these national surveys [41, 47], the racial/ethnic differences in Latent Class 1 membership and higher intentions for Latent Class 1 to engage in COVID-19 preventive behaviors may be partially explained by higher perceived COVID-19 threat among students from minoritized racial/ethnic groups and lower perceived threat among Non-Hispanic White students. As noted in previous literature, one key takeaway from these findings may be a need to emphasize the importance of COVID-19 preventive behaviors among those with lower perceived COVID-19 threat. In many cases, this emphasis may be particularly important for Non-Hispanic White students who may perceive lower COVID-19 threat due to lower national rates of COVID-19 infection, hospitalization, and mortality.

In addition, compared to the reference group, there were higher intentions to social distance among students in Latent Class 1, but non-significant differences for mask-wearing and vaccination. Perhaps students in Latent Class 1, who had a higher probability of experiencing personal healthcare discrimination, may have been more likely to social distance because social distancing offered protection from COVID-19 with few costs (e.g., money, time) and did not require interaction with the healthcare system. Alternatively, differences in social distancing based on latent classes may have been easier to observe than the other COVID-19 preventive behaviors given that there were UMD campus mandates to wear a mask and receive the COVID-19 vaccine (i.e., students may have answered relatively high on the intention to wear a mask and receive the COVID-19 vaccine, potentially concealing some of the expected differences between latent classes).

Moreover, the lack of a difference in vaccination intentions for students in Latent Class 1 compared to the reference group was distinct from much of the existing literature on racial/ethnic differences in vaccine hesitancy in the general population [48] as well as college students [49]. These findings were also somewhat contrary to the a priori expectations that perceived U.S. healthcare system inequities and personal experiences of healthcare discrimination would be associated with lower preventive health behaviors, especially COVID-19 vaccination which requires interaction with the healthcare system. It is possible, however, that the campus setting reduced disparities in healthcare access that otherwise exist in the national population, as COVID-19 vaccines were easily accessible on UMD’s campus. In addition, it is also possible that higher COVID-19 threat observed among members of Latent Class 1 may have outweighed their concerns about U.S. healthcare system inequities and anticipated healthcare discrimination. Furthermore, despite study findings that students from Latent Class 1 had high intentions to engage in COVID-19 preventive behaviors, racial/ethnic disparities in COVID-19 outcomes persist among college students in the broader population. This may suggest that behavioral intentions cannot fully mitigate structural factors that influence students’ ability to engage in COVID-19 preventive behaviors. There may be cases, for example, in which students adhere to COVID-19 preventive measures, but are still at high risk of COVID-19 infection (e.g., close living or working conditions when social distancing is more difficult and/or mask-wearing is less effective).

### Limitations

Several limitations in the present study should be considered when interpreting the study findings. Given that participants were recruited from one university and the relatively small sample size (*N* = 432), the study findings may not be representative of all college students in the U.S. (e.g., limited representation across all racial/ethnic groups and gender identities). Participants who selected their race as “Other” and those who identified their gender identity as transgender, gender non-conforming, or who selected gender as “not listed” were excluded from the study due to small sample sizes. Further, the high proportion of female participants (75.0%) in the study population could have affected responses to the COVID-19 preventive behavioral intentions given prior evidence that females reported engaging in more COVID-19 preventive measures than males due to greater fear of the COVID-19 pandemic [50]. Moreover, in the present study, no students identified as Native Hawaiian or Pacific Islander, or American Indian or Alaska Native and a larger proportion of students identified as Non-Hispanic Asian (20.6%) compared to those who identified as Asian (6.7%) among all undergraduate college students in the U.S [51]. In addition, the smaller sample size also limited the ability to interpret findings related to Latent Class 3, which only included a subgroup of 22 participants. Furthermore, given that this study did not include random selection and participants self-selected into the study, students who participated in the study may have been more interested in health topics than the general student population (e.g., students who majored in public health may have been overrepresented in the study sample as students were not asked to identify their major in the survey). This type of recruitment may have introduced selection bias and could have contributed to the relatively high perceived U.S. healthcare system inequities in most of the study sample. The study sample, however, was representative of the larger university’s undergraduate population by race/ethnicity [52] (i.e., Non-Hispanic White (49.5% in the present study versus 45.0% in the university), Non-Hispanic Asian (20.6% versus 20.3%), Hispanic or Latino (11.6% versus 10.2%), Non-Hispanic Black or African American (11.3% versus 11.8%), Non-Hispanic Multiracial (6.9% versus 4.7%)). While the study focused on college students, it should be noted that college students, regardless of race/ethnicity, often have greater access to resources (e.g., disease prevention education, easier access to COVID-19 testing) than their non-student peers. This could therefore affect the generalizability of the study results to young adults who do not attend college. Further, given that fewer preventive behaviors were observed among individuals with less education [53], socioeconomic factors may impact COVID-19 related perceptions, creating potential interactions between these factors and their association with students’ preventive behavioral intentions. Lastly, the study was conducted over a relatively long period of time (i.e., December 2020-December 2021). Survey completion date was included as a covariate to help mitigate this potential limitation, yet few participants completed the survey during the middle stages (i.e., months 5–8) of data collection. This limited the ability to capture health-related perceptions and COVID-19 preventive behavioral intentions during this period and potential fluctuating patterns in these relationships.

### Strengths

Despite these limitations, the study has several strengths. The analysis was particularly well-suited for identifying subgroups of individuals given the ability of latent class analyses to perform well in sample sizes of 300 participants or more [54]. The analysis also included three different measures of COVID-19 preventive behavioral intentions, which allowed for examining whether the influence of latent class health-related perceptions on COVID-19 behavioral intentions varied across the three different measures. In addition, the use of latent class indicators related to perceived U.S. healthcare system inequities and personal experiences of healthcare discrimination extends previous health belief models that only focused on perceived COVID-19 threat [55]. This extension suggests an interconnected nature of complex health-related perceptions across domains of individual risk and the healthcare system and presents opportunities to further explore potential interactions between these domains. It is possible, for example, that perceived U.S. healthcare system inequities and personally experienced healthcare discrimination may moderate the relationship between perceived COVID-19 threat and COVID-19 preventive behavioral intentions. Future studies could consider investigating these relationships.

### Implications and conclusions

The present study has implications for messaging campaigns to increase adherence to COVID-19 preventive behavioral interventions among college students in the U.S., particularly for students who perceive lower COVID-19 threat. These campaigns may need to emphasize the importance of COVID-19 prevention regardless of whether a student feels they are personally at risk. Participating in these preventive behaviors contributes to a safe student community, thus, framing these behaviors as a social responsibility may increase preventive behavioral intentions even if the low perception of susceptibility remains, which consequently could improve the pandemic response. Additionally, this study has implications for interventions targeting racial/ethnic disparities in COVID-19 outcomes. Given that intentions to engage in COVID-19 preventive behaviors may already be high among students from minoritized racial/ethnic groups, interventions could consider focusing efforts on increasing access to COVID-19 vaccines and/or masks and helping to address environmental factors that increase COVID-19 risk despite adherence to COVID-19 preventive behaviors (e.g., addressing structural factors and exposure related to living or work conditions). Lastly, future studies could examine whether higher concerns over COVID-19 paired with low confidence in equitable treatment from the healthcare system compromises college students’ mental and physical health, particularly for students from minoritized racial/ethnic backgrounds. This study has lasting implications even as COVID-19 preventive guidelines continue to evolve. Understanding college students’ perceptions of recommended preventive behaviors and their experiences and/or perceptions of the U.S. healthcare system can inform efforts to improve the health of college students and may inform responses to future pandemics. The present study findings and implications for COVID-19 messaging campaigns, interventions to reduce COVID-19 risk, and future research on racial/ethnic differences in college students’ mental and physical health related to COVID-19 concerns can continue to inform responses to the COVID-19 outbreak as well as potential future pandemics.

## Supplementary Information

**Additional file 1: Table S1. **Latent class indicators. **Table S2. **COVID-19 preventive behavior scenarios. **Table S3. **Model fit indices used in model selection. **Table S4**. Associations between perceived COVID-19 threat, perceived U.S. healthcare system inequities, personal experiences of healthcare discrimination and COVID-19 preventive behavioral intentions. **Figure S1.** 4-class solution of latent classes. **Figure S2. **Likelihood of COVID-19 preventive behavioral intentions*.

## Data Availability

The datasets used and/or analyzed during the current study are available from the corresponding author upon request.
