# Supplementary material for: Perceived COVID-19 threat, perceived healthcare system inequities, personal experiences of healthcare discrimination and their associations with COVID-19 preventive behavioral intentions among college students in the U.S
Source: BMC Public Health. 2022 Dec 31;22:2458. doi: 10.1186/s12889-022-14438-5 (PMC9803883; doi:10.1186/s12889-022-14438-5)
Supplement: Supplementary file 1 — Additional file 1: Table S1. Latent class indicators. Table S2. COVID-19 preventive behavior scenarios. Table S3. Model fit indices used in model selection. Table S4. Associations between perceived COVID-19 threat, perceived U.S. healthcare system inequities, personal experiences of healthcare discrimination and COVID-19 preventive behavioral intentions. Figure S1. 4-class solution of latent classes. Figure S2. Likelihood of COVID-19 preventive behavioral intentions*. [file 12889_2022_14438_MOESM1_ESM.docx]

**Perceived COVID-19 threat, perceived healthcare system inequities, personal experiences of healthcare discrimination and their associations with COVID-19 preventive behavioral intentions among college students in the U.S.**

**Supplemental Material**

**Table S1. Latent class indicators**

| **Items** | **Responses** |
| --- | --- |
| Perceived COVID-19 Threat |  |
| 1. You believe COVID-19 is serious and life threatening (perceived severity) | 1. Very true 2. Somewhat true 3. Not true |
| 1. You are concerned about contracting COVID-19 (COVID-19 susceptibility) | 1. Very true 2. Somewhat true 3. Not true |
| Perceived U.S. Healthcare System Inequities |  |
| 1. How often have racial and ethnic minority patients with COVID-19 been treated unfairly by the U.S. healthcare system because of their race or ethnicity? (Treatment of COVID-19 patients) | 1. Very often 2. Somewhat often 3. Never |
| 1. How true is it that racial and ethnic minority groups have less access to COVID-19 testing compared to Whites? (Access to COVID-19 testing) | 1. Very true 2. Somewhat true 3. Not true |
| 1. How confident are you that the COVID-19 vaccine will be distributed fairly across racial and ethnic groups? (COVID-19 vaccine distribution) | 1. Not confident 2. Somewhat confident 3. Very confident |
| Personal Experiences of Healthcare Discrimination |  |
| 1. When getting healthcare, how often has each experience happened to you because of your race or skin color?  - Treated with less courtesy than other people - Treated with less respect than other people - Received poorer services than other people - Had a doctor or nurse act as if he or she thought you were not smart - Had a doctor or nurse act as if he or she was afraid of you - Had a doctor or nurse act as if he or she was better than you - Felt like a doctor or nurse was not listening to what you were saying | 1. Never 2. Once 3. 2-3 times 4. 4 times or more |

**Table S2. COVID-19 preventive behavior scenarios**

| **COVID-19 Preventive Behavior** | **Scenario** | **Response Options** |
| --- | --- | --- |
| Social Distancing | Imagine that you need to go out in public. Public health guidelines advise you to social distance (remain 6 feet apart from others). How likely are you to social distance in public? | -Extremely unlikely  -Moderately unlikely  -Slightly unlikely  -Slightly likely  -Moderately likely  -Extremely likely |
| Mask-Wearing | Imagine that you need to go out in public. Public health guidelines advise you to wear a mask. How likely are you to wear your mask in public? | -Extremely unlikely  -Moderately unlikely  -Slightly unlikely  -Slightly likely  -Moderately likely  -Extremely likely |
| COVID-19 Vaccination | Imagine you are at the doctor's office and you are offered the COVID-19 vaccine. It has gone through clinical trials and been approved by the FDA and World Health Organization. The doctor explains the following information: In general, vaccines are often effective at preventing certain illnesses. There is a small chance that the vaccine itself makes some individuals feel some of the symptoms associated with the illness itself (such as fatigue, cough, fever, and chest pain). How likely are you to get the COVID-19 vaccine? | -Extremely unlikely  -Moderately unlikely  -Slightly unlikely  -Slightly likely  -Moderately likely  -Extremely likely |

**Table S3. Model fit indices used in model selection**

|  | **SA-BIC** | **Entropy** | **LMR**  **LRT** | **p-**  **value** | **PB**  **LRT** | **p-**  **value** |
| --- | --- | --- | --- | --- | --- | --- |
| **1-Class Solution** | 2821.09 | - | - | - | - | - |
| **2-Class Solution** | 2662.66 | 0.72 | 175.20 | 0.00 | 179.26 | <0.01 |
| **3-Class Solution** | 2644.00 | 0.74 | 38.61 | 0.00 | 39.50 | <0.01 |
| **4-Class Solution** | 2642.75 | 0.81 | 21.58 | 0.06 | 22.08 | <0.01 |
| **5-Class Solution** | 2649.66 | 0.86 | 13.62 | 0.19 | 13.94 | 0.23 |

SA-BIC: sample-size adjusted Bayesian Information Criterion; LMR LRT: Lo-Mendell Rubin likelihood ratio test; PB LRT: parametric bootstrapped likelihood ratio test. Stronger model fit indicated by lower SA-BIC, higher entropy, and p-values <0.05 for the likelihood ratio tests.

**Table S4. Associations between perceived COVID-19 threat, perceived U.S. healthcare system inequities, personal experiences of healthcare discrimination and COVID-19 preventive behavioral intentions**

|  | **Estimate** | **Standard Error** | **p value** |
| --- | --- | --- | --- |
| **Social Distancing** | | | |
| Perceived COVID-19 Severity (Very severe vs. somewhat or not severe) | 1.10 | 0.15 | <0.01 |
| Perceived COVID-19 susceptibility (Very susceptible vs somewhat to not susceptible) | 0.89 | 0.13 | <0.01 |
| Perceptions of the U.S. healthcare system (Never, once, 2-3 times, 4 times or more) | 0.21 | 0.15 | 0.15 |
| Perceived fairness in treatment of racial and ethnic minority patients with COVID-19 (Never vs. somewhat to very often) | 0.63 | 0.21 | <0.01 |
| Perceived access to COVID-19 testing for racial and ethnic minority groups compared to White individuals (Not true vs. somewhat to very true) | 0.39 | 0.17 | 0.02 |
| Perceived distribution of the COVID-19 vaccine across racial and ethnic groups (Very confident vs. somewhat to not confident) | 0.60 | 0.19 | <0.01 |
| **Mask-Wearing** | | | |
| Perceived COVID-19 Severity (Very severe vs. somewhat or not severe) | 0.68 | 0.12 | <0.01 |
| Perceived COVID-19 susceptibility (Very susceptible vs somewhat to not susceptible) | 0.51 | 0.10 | <0.01 |
| Perceptions of the U.S. healthcare system (Never, once, 2-3 times, 4 times or more) | 0.06 | 0.12 | 0.59 |
| **Mask-Wearing** | | | |
| Perceived fairness in treatment of racial and ethnic minority patients with COVID-19 (Never vs. somewhat to very often) | 0.71 | 0.16 | <0.01 |
| Perceived access to COVID-19 testing for racial and ethnic minority groups compared to White individuals (Not true vs. somewhat to very true) | 0.56 | 0.13 | <0.01 |
| Perceived distribution of the COVID-19 vaccine across racial and ethnic groups (Very confident vs. somewhat to not confident) | 0.75 | 0.15 | <0.01 |
| **COVID-19 Vaccination** | | | |
| Perceived COVID-19 Severity (Very severe vs. somewhat or not severe) | 0.40 | 0.12 | <0.01 |
| Perceived COVID-19 susceptibility (Very susceptible vs somewhat to not susceptible) | 0.10 | 0.10 | 0.35 |
| Perceptions of the U.S. healthcare system (Never, once, 2-3 times, 4 times or more) | -0.29 | 0.11 | 0.11 |
| Perceived fairness in treatment of racial and ethnic minority patients with COVID-19 (Never vs. somewhat to very often) | 0.48 | 0.16 | <0.01 |
| Perceived access to COVID-19 testing for racial and ethnic minority groups compared to White individuals (Not true vs. somewhat to very true) | 0.58 | 0.13 | <0.01 |
| Perceived distribution of the COVID-19 vaccine across racial and ethnic groups (Very confident vs. somewhat to not confident) | 0.40 | 0.15 | <0.01 |

**Figures**

**Figure S1: 4-class solution of latent classes**


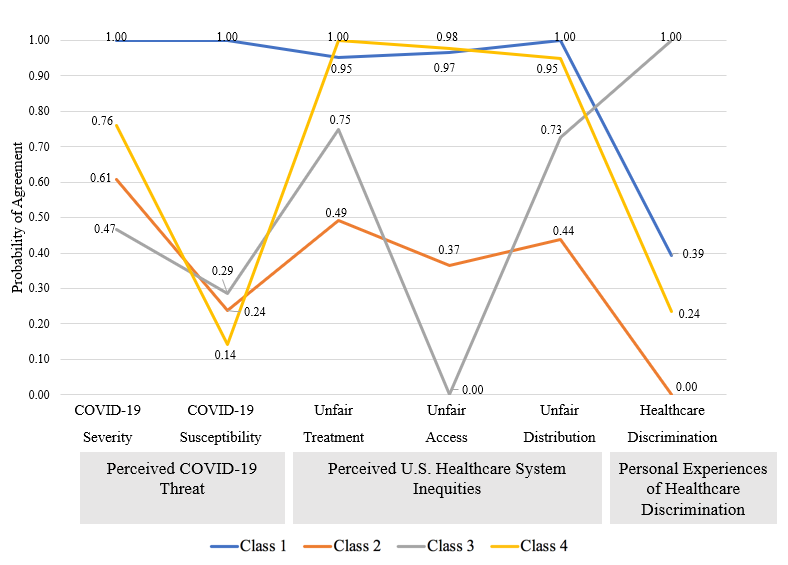


**Figure S2: Likelihood of COVID-19 preventive behavioral intentions***


*Likelihood of COVID-19 preventive behavioral intentions represents predicted value of intercept, adjusting for age, gender, household income, and survey completion date.
